# Isolation of a *Yersinia enterocolitica* biotype 1B strain in France, and evaluation of its genetic relatedness to other European and North American biotype 1B strains

**DOI:** 10.1038/s41426-018-0123-0

**Published:** 2018-07-04

**Authors:** Cyril Savin, Anne-Sophie Le Guern, Matthieu Lefranc, Sylvie Brémont, Elisabeth Carniel, Javier Pizarro-Cerdá

**Affiliations:** 1 Yersinia Research Unit/Yersinia National Reference Laboratory/WHO Collaborating Center for Yersinia, Institut Pasteur, 75015 Paris, France; 2 CBM25 – Laboratoire de biologie médicale de terre rouge, 25000 Besançon, France

*Yersinia enterocolitica*, a member of the *Enterobacteriaceae* family, is a gastrointestinal pathogen. This species is transmitted via the fecal-oral route, usually through the consumption of contaminated food, and less often through direct contact with infected animals^1^. *Y. enterocolitica* infections cause diarrhea, fever, and abdominal pain, mimicking appendicitis, and predominate in children^2^. The species *Y. enterocolitica* is subdivided into six biotypes: the nonpathogenic biotype 1A, low-pathogenicity biotypes 2–5, which cause self-limiting infections, and the highly pathogenic biotype 1B, which is more commonly associated with systemic dissemination^3,4^.

While biotype 4 is now predominant in North America^1,5^, biotype 1B was responsible for most of the *Y. enterocolitica* infections in the USA until the end of the 1980s^5,6^. The first outbreak due to *Y. enterocolitica* 1B was reported in the state of New York, USA, in 1976, with more than 200 children and school employees infected after the consumption of contaminated chocolate milk, resulting in hospitalization of 36 children^7^. Other outbreaks were reported in 1981, 1982, and 1995, due to the consumption of powder milk, tofu, or Pasteurized milk^1^.

*Y. enterocolitica* biotype 1B strains have rarely been isolated in Europe. In the past 20 years, only two clinical strains of this biotype have been isolated in France among a total of 9442 *Yersinia* strains characterized by the French National Reference Laboratory, with the last one, isolated from a clinical cutaneous abscess, dating to 2004 (unpublished data). In Italy, five biotype 1B strains were isolated between 1979 and 1989^8^, and in Germany, the first isolation was reported in 2002^9^, but no isolations have been reported in these countries since then. On the other hand, the first *Y. enterocolitica* 1B strain in Poland was isolated in 2004 from a patient who suffered from liver cirrhosis^10^, and the number of isolated strains has dramatically increased since then^11,12^. Owing to the systemic dissemination potential of *Y. enterocolitica* 1B strains, their spread may cause a serious public health threat, thus rendering their surveillance crucial.

Herein, we report the case of *Y. enterocolitica* 1B infection in a French patient, as well as the characterization of the strain and genomic analysis of its genetic relatedness to other biotype 1B strains.

## The case

Here, we report the case of a French 42-year-old woman who presented with chronic diarrhea, abdominal pain, and vomiting in August 2017. No history of travel outside metropolitan France in the last 6 months was reported. She was treated with ciprofloxacin for 7 days. Subsequently, the patient developed inflammatory bowel disease, and a colonoscopy revealed terminal ileitis, a hallmark of enteric yersiniosis^13^. Collected stools were semiliquid and glairy, and their microscopic examination revealed the presence of rare leukocytes but no erythrocytes, suggesting a bacterial infection. A stool sample was seeded on cefsulodin irgasan novobiocin (CIN) agar medium (Biomérieux, Cat. #43421, Craponne, France), and incubated for 48 h at 30 °C. Few *Yersinia*-like colonies grew, and a *Y. enterocolitica* strain was identified by mass spectrometry (MALDI-TOF, Bruker Microflex, BDD 6903 MSP) with a score of 2.37. The strain was sent to the French *Yersinia* National Reference Laboratory (Institut Pasteur, Paris, France), where tests using API20E and 50CH strips, Tween-esterase, pyrazinamidase activity, and O-antigen seroagglutination revealed that the strain (IP39285) belonged to the *Y. enterocolitica* bioserotype 1B/O:7,8-8-8,19. Since isolation of biotype 1B strains is extremely rare in France, polymerase chain reaction (PCR), targeting *yadA* (located on the *Yersinia* virulence plasmid) and *fyuA* (located in the *Yersinia* high-pathogenicity island), was performed. Both PCR tests were positive, confirming that IP39285 was a highly pathogenic biotype 1B strain. Multilocus sequence analysis (*glnA*, *gyrB*, *hsp60,* and *recA*)^14^ showed that this strain belonged to the same branch as other biotype 1B strains (data not shown). Antimicrobial susceptibility testing, based on the 2017 EUCAST guidelines, revealed resistance of IP39285 to amoxicillin, as usually observed for *Y. enterocolitica* 1B strains, and its susceptibility to all other antibiotics tested (amoxicillin-clavulanic acid, cephalexin, cefoxitin, ceftriaxone, ciprofloxacin, nalidixic acid, trimethoprim, sulfonamide, tetracycline, and ticarcillin).

We next evaluated the genetic relatedness of IP39285 to three other biotype 1B strains of French, Belgian, and USA origin previously isolated at the *Yersinia* National Reference Laboratory, and to 16 biotype 1B strains of Belgian, German, USA and unknown origin, whose genomic sequences are publicly available (see Supplementary Table). Whole-genome sequencing of strains IP39285, IP28308, IP35698, and IP38642 was performed using a NextSeq® 500 sequencing system (Illumina, San Diego, CA, USA). The annotated genome of the IP39285 strain was deposited to GenBank, and is available under BioProject PRJNA407758 (accession number NWMR00000000). The genome was annotated with the NCBI Prokaryotic Genome Annotation Pipeline (PGAP), and consists of 4,325,608 bp in 143 contigs, with a G + C content of 47.5%. PGAP found a total of 4061 genes, 3988 coding sequences (CDSs), 73 RNA genes, and 169 pseudogenes. The G + C content and the number of CDSs are similar to those of other publicly available genomes of biotype 1B *Y. enterocolitica* strains. In silico genomic analysis revealed the presence of classic virulence genes, including *ail*, *inv*, *ystA,* and *myfA*, located on the chromosome; *fyuA* and *irp2*, located in the *Yersinia* high-pathogenicity island; and *yadA* and *virF*, located on the *Yersinia* virulence plasmid. Prediction of antimicrobial susceptibility genes in the IP39285 genome using ResFinder (http://www.genomicepidemiology.org/) allowed the identification of the *blaA* gene, responsible for beta-lactam resistance, as in all other biotype 1B strains, and of the *vatF* gene, responsible for streptogramin B resistance, as in 14 out of the 18 other biotype 1B genomes tested.

**Fig. 1:**
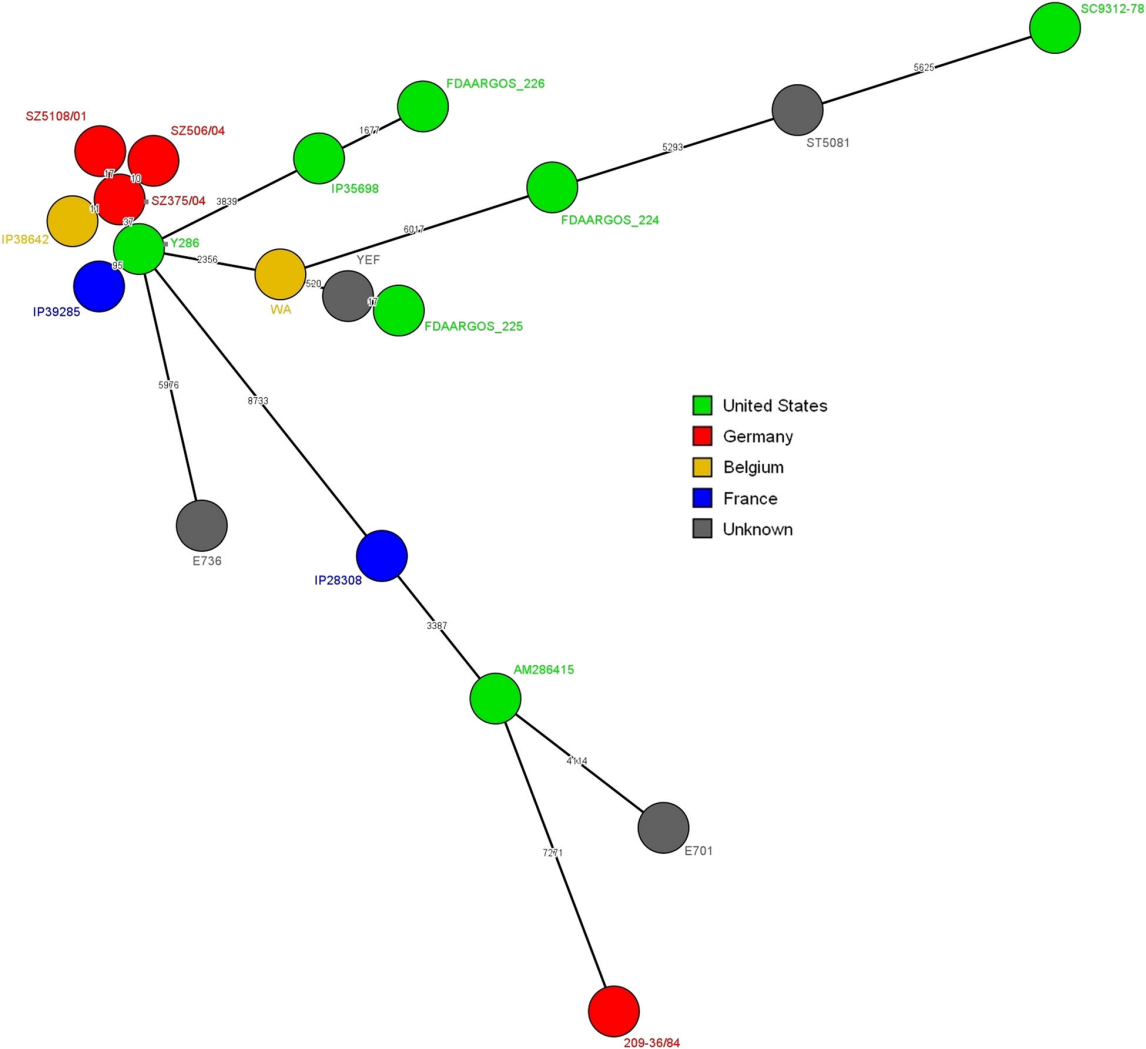
A minimal spanning tree of 19 *Y. enterocolitica* biotype 1B strains compared with wgSNP analysis using Bionumerics 7.6 (Applied Maths, Sint-Martens-Latem, Belgium). Numbers on the branches indicate the difference in SNPs between two strains. Logarithmic scaling was used for branch lengths

We then conducted a comparative genomic analysis to identify single-nucleotide polymorphisms (SNPs) with the wgSNP analysis module of Bionumerics 7.6 (Applied Maths, Sint-Martens-Latem, Belgium) using the genome of strain 8081 as a reference (accession number AM286415) (Fig. 1). In total, 25,671 SNP positions were identified in the 19 compared strains, highlighting a high genetic diversity among these isolates (Fig. 1). For comparison, in 264 *Y. enterocolitica* biotype 4 strains with no epidemiological links isolated worldwide in the past 30 years, only 9827 SNPs were identified (data not shown). This diversity was also observed using a pairwise comparison with numerous branches, whose length was more than 1000 SNPs. Despite this diversity, we identified a cluster of six highly homogeneous strains (maximum difference of approximately 100 SNPs), which included IP39285. Within this cluster, a Belgian strain, IP38642, and German strains, SZ506/04, SZ375/34, and SZ5108/01, had fewer than 18 SNPs, indicating that the strains were highly related to each other. The USA strain Y286 had only a 37-SNP difference from the German strain SZ375/04, despite their geographical distance. The closest relative to the French strain IP39285 was the Y286 strain, with a 95-SNP difference. Other European and North American strains were distant from the Y286 strain, with at least 2356 SNP difference.

Altogether, analysis of the cluster including strain IP39285 and of the other, more distant branches suggested potential circulation of *Y. enterocolitica* biotype 1B strains between North America and Europe. We were also able to detect the microevolution of a *Y. enterocolitica* biotype 1B clone settled in Europe, including Belgian and German strains. Considering the recent increase in *Y. enterocolitica* biotype 1B infections in some European countries, our results highlight the need for more sustained surveillance of this highly pathogenic strain, whose spread may pose a global public health threat.

## Electronic supplementary material

Supplementary Table S1
