# Supplementary material for: Isolation of a Yersinia enterocolitica biotype 1B strain in France, and evaluation of its genetic relatedness to other European and North American biotype 1B strains
Source: Emerg Microbes Infect. 2018 Jul 4;7:121. doi: 10.1038/s41426-018-0123-0 (PMC6030111; doi:10.1038/s41426-018-0123-0)
Supplement: Supplementary file 1 — Supplementary Table S1 [file 41426_2018_123_MOESM1_ESM.docx]

Supplementary Table: Characteristics of the studied *Y. enterocolitica* biotype 1B strains

| Strain number | Serotype | Country | Host | Origin | Isolation date | Accession number |
| --- | --- | --- | --- | --- | --- | --- |
| IP39285 | O:7,8-8-8,19 | France | Human | Stool | 2017 | This study |
| IP38642 | O:7,8-8-8,19 | Belgium | Human | Stool | 2017 | This study |
| IP35698 | O:7,8-8-8,19 | USA | Human | Stool | 2011 | This study |
| IP28308 | O:21 | France | Human | Cutaneous abscess | 2004 | This study |
| E736 | O:21 | UN | Human | Stool | UN | ERR163911 |
| SC9312-78 | O:13,18 | USA | Human | UN | UN | ERR163913 |
| ST5081 | UN | UN | UN | UN | UN | ERR163912 |
| WA | O:8 | Belgium | Human | Blood | UN | ERR163909 |
| Y286 | O:8 | USA | UN | UN | UN | ERR024887 |
| 209-36/84 | O:21 | Germany | Human | UN | UN | ERR024601 |
| SZ375/04 | O:8 | Germany | Human | UN | UN | ERR024884 |
| SZ506/04 | O:8 | Germany | Human | UN | UN | ERR024889 |
| SZ5108/01 | O:8 | Germany | Human | UN | UN | ERR024600 |
| E701 | O:4,32 | UN | Human | Stool | UN | ERR163910 |
| 8081 | O:8 | USA | Human | Blood | UN | AM286415 |
| FDAARGOS_224 | UN | USA | Monkey | Tissue | 1970 | SRR4272709 |
| FDAARGOS_225 | O:8 | USA | Human | Facial abscess | 1934 | SRR4273027 |
| FDAARGOS_226 | O:8 | USA | Human | Fluid from anterior chamber of the eye | 1968 | SRR4273029 |
| YEF | UN | UN | UN | UN | UN | SRR2180266 |

UN : unknown
